# Reducing the Risk of Transmission of Critical Antimicrobial Resistance Determinants From Contaminated Pork Products to Humans in South-East Asia

**DOI:** 10.3389/fmicb.2021.689015

**Published:** 2021-07-27

**Authors:** Wandee Sirichokchatchawan, Prasert Apiwatsiri, Pawiya Pupa, Imporn Saenkankam, Nwai Oo Khine, Angkana Lekagul, Kittitat Lugsomya, David J. Hampson, Nuvee Prapasarakul

**Affiliations:** ^1^College of Public Health Sciences, Chulalongkorn University, Bangkok, Thailand; ^2^Diagnosis and Monitoring of Animal Pathogen Research Unit, Chulalongkorn University, Bangkok, Thailand; ^3^Department of Veterinary Microbiology, Faculty of Veterinary Science, Chulalongkorn University, Bangkok, Thailand; ^4^International Health Policy Program, Ministry of Public Health, Nonthaburi, Thailand; ^5^Jockey Club College of Veterinary Medicine and Life Sciences, City University of Hong Kong, Kowloon Tong, Hong Kong; ^6^School of Veterinary Medicine, Murdoch University, Perth, WA, Australia

**Keywords:** antibiotic resistance, alternatives to antibiotics, one-health, pig production, policy, slaughtering process, South East Asia

## Abstract

Antimicrobial resistance (AMR) is a critical challenge worldwide as it impacts public health, especially via contamination in the food chain and in healthcare-associated infections. In relation to farming, the systems used, waste management on farms, and the production line process are all determinants reflecting the risk of AMR emergence and rate of contamination of foodstuffs. This review focuses on South East Asia (SEA), which contains diverse regions covering 11 countries, each having different levels of development, customs, laws, and regulations. Routinely, here as elsewhere antimicrobials are still used for three indications: therapy, prevention, and growth promotion, and these are the fundamental drivers of AMR development and persistence. The accuracy of detection of antibiotic resistant bacteria (ARB) and antibiotic resistance genes (ARG) depends on the laboratory standards applicable in the various institutes and countries, and this affects the consistency of regional data. *Enterobacteriaceae* such as *Escherichia coli* and *Klebsiella pneumoniae* are the standard proxy species used for indicating AMR-associated nosocomial infections and healthcare-associated infections. Pig feces and wastewater have been suspected as one of the hotspots for spread and circulation of ARB and ARG. As part of AMR surveillance in a One Health approach, clonal typing is used to identify bacterial clonal transmission from the production process to consumers and patients – although to date there have been few published definitive studies about this in SEA. Various alternatives to antibiotics are available to reduce antibiotic use on farms. Certain of these alternatives together with improved disease prevention methods are essential tools to reduce antimicrobial usage in swine farms and to support global policy. This review highlights evidence for potential transfer of resistant bacteria from food animals to humans, and awareness and understanding of AMR through a description of the occurrence of AMR in pig farm food chains under SEA management systems. The latter includes a description of standard pig farming practices, detection of AMR and clonal analysis of bacteria, and AMR in the food chain and associated environments. Finally, the possibility of using alternatives to antibiotics and improving policies for future strategies in combating AMR in a SEA context are outlined.

## Introduction

Antimicrobial resistance (AMR) in bacterial species and strains is a critical global threat, and has been monitored in animals, humans and the environment through international collaborations aimed at AMR reduction (Gerbin, 2014; Lechner et al., 2020; Singh et al., 2021). The main factors contributing to the emergence of AMR are overuse and misuse of antimicrobials, and the lack of quality control of antimicrobials and active pharmaceutical ingredients in the market (Simba et al., 2016; Tangcharoensathien et al., 2018; Barroga et al., 2020). Also, the multi-faceted nature of the problem includes misconceptions about antibiotic types and AMR; lack of diagnostic facilities; limited availability of alternatives to antibiotics as a means to establish antibiotic-free farms; and insufficient training of veterinarians on AMR and antibiotic prescribing (Lekagul et al., 2021). Low- and middle-income countries (LMICs), including those in South East Asia (SEA) are crucial in the global response to AMR due to their diverse medicine regulation and access to antimicrobials. Furthermore, folk conceptions or unexpected practices around antimicrobials in these regions can complicate AMR communication efforts and entail unforeseen consequences (Haenssgen et al., 2019). Nevertheless, it is encouraging that countries such as Denmark and the Netherlands that both have massive pig production in recent years have achieved tremendous reductions in antimicrobial usage while sustaining peak production (Dall, 2019). Comparable results have been accomplished in Belgium, France, Sweden, and the United Kingdom (More, 2020). In Brazil, one of the biggest pork producers in the world, the increased demand for meat production has led to ongoing environmental problems, such as soil and water contamination by pathogenic and/or resistant microorganisms (Silva et al., 2015; Brisola et al., 2019). In Russia, relatively high levels of MDR *Escherichia coli* that are resistant to critically important antimicrobials such as colistin, cefotaxime, and ciprofloxacin have been recorded (Makarov et al., 2020). A study in New Zealand also found that the widespread use of oral antimicrobials within pig production was a significant risk factor for development of AMR and MDR *E. coli* (Riley et al., 2020). The rate of antibiotic resistance differs considerably from country to country, depending upon the amount of usage. In the EU, the lowest levels of AMR *E. coli* isolates were found in countries where lower antimicrobial usage was practiced, such as in Norway, Sweden, and Finland, whereas countries with high levels of use such as Spain, Portugal, and Belgium had relatively higher levels of AMR *E. coli* (Holmer et al., 2019).

Nosocomial infections or healthcare-associated infections fall under the political concerns of the World Health Organization (WHO). Nosocomial infections account for 7 and 10% of all infections in developed and developing countries, respectively (Khan et al., 2017; World Health Organization [WHO], 2019). *Enterobacteriaceae* such as *Escherichia coli* and *Klebsiella pneumoniae* have been identified as the most common resistant bacteria found in healthcare-associated infections (Malchione et al., 2019; Shrestha et al., 2019). The possibility that AMR colonization in humans can be related to livestock and environmental sources was shown in a recently published One Health AMR risk assessment (Opatowski et al., 2020). WHO emphasizes the importance of antimicrobial classes (agents) that are used in humans being targeted for antimicrobial susceptibility surveillance in livestock, including aminoglycosides (gentamicin), carbapenems (meropenem), cephalosporins (3rd, 4th, and 5th generation: ceftriaxone, cefepime, ceftaroline, ceftobiprole), glycopeptides (vancomycin), amoxicillin-clavulanic-acid, polymyxins (colistin), and quinolones (ciprofloxacin). Due to high prevalence and incidence of AMR rates in the pig industries in South-East Asian (ASEAN) countries, a regional database of AMR surveillance has been developed (Nguyen N. T. et al., 2016; Nhung et al., 2016; Lugsomya et al., 2018a, b; Mobasseri et al., 2019; Vounba et al., 2019; Khine et al., 2020; World Health Organization [WHO], 2020; Dawangpa et al., 2021). It is important to emphasize that SEA is a diverse region, where different levels of development, customs, laws, and regulations among different countries in the region. Pork is one of the most common protein sources and is widely produced for local consumption, with some export also occurring. Hence it is an important target for AMR surveillance.

Antimicrobial resistance surveillance through the pig production cycle, covering from the neonatal to the slaughtering periods has demonstrated the possibility of transmission of AMR bacteria in the food chain, and has provided a modicum of comparative evidence for nosocomial infection through genetic fingerprinting of isolates using multi-locus sequence typing (MLST) and whole-genome sequencing (Mather et al., 2018). A plasmid carrying *mcr-1* conferring colistin resistance was first identified in China in 2014 (Liu et al., 2016). To date, up to 10 types of the *mcr* gene family harbored in *Enterobacteriaceae* have been reported in clinical samples and in animals and human carriers (Xu et al., 2021). Colistin has been extensively used in pig production, often as a growth promoter until the 2017 prohibition of its use under regional policy. However, a high prevalence of colistin-resistant *Klebsiella pneumoniae* was found in healthy people living in rural communities in Thailand, Vietnam, and Laos with no history of colistin therapy (Olaitan et al., 2014; Yamaguchi et al., 2020). A 10–22% prevalence of the *mcr-1* and *mcr-3* genes has been reported in *E. coli* isolated from pig and chicken farms in Vietnam and Thailand (Malhotra-Kumar et al., 2016; Khine et al., 2020) and in *Klebsiella pneumoniae* from farmed pigs in Malaysia (Mobasseri et al., 2019). The clonal similarity of a colistin-resistant *E. coli* isolate from a local pig with a human isolate was demonstrated in Laos (Olaitan et al., 2015). Overall, these occurrences confirm the interchangeability of colistin-resistant bacteria between humans and farm animals that use colistin as a feed additive. Cross-species transmission is a suspected course of distribution.

Similar clonal types of multidrug-resistant *Salmonella* Typhimurium and *E. coli* have been found in humans and pigs in SEA and in the United States (Oloya et al., 2009; Nhung et al., 2016; Mather et al., 2018; Sudatip et al., 2021). Pigs and the environment can be potential sources for the dissemination of AMR to humans; however, there have been a few longitudinal studies monitoring linkages of AMR strains among pigs, the environment, and consumers/patients. Genetic similarity between bacterial clones from farmed pigs and pork was not found in any comprehensive monitoring study in Thailand (Lugsomya et al., 2018b). To our knowledge, to date there has been no unequivocal direct evidence of transmission of AMR bacteria to human patients from farms or the slaughtering process in SEA.

This article aims to improve awareness and understanding of AMR through a description of the occurrence of AMR in pig farm and food chains under AMR management systems in SEA: this includes a description of standard farming practices; detection of AMR and clonal analysis of bacteria; AMR in the food chain, wastewater, and associated environments, the possibilities of AMR transfer from farming and slaughtering practices to humans. Finally, the possibility of reducing AMR by using alternatives to antibiotics and improving policies are discussed.

## Standard Farming Systems in Sea

Asia is the largest producer of pigs in the world (58.4%), and within SEA the top three pig producing countries are Myanmar, Vietnam, and the Philippines. In contrast, Thailand exports the most pork raised under a high standard of farming and food safety conditions (FAOSTAT, 2019). Pig production systems in SEA vary greatly, from backyard pigs held in small-scale often peri-urban farms to semi-commercial units and large intensive units. For example, 80% of pig farms in Cambodia involve smallholders, while 80% in Thailand are intensive farms. However, even pig farming in peri-urban areas operates under a high standard of biosecurity similar to that in large commercial approved farms (World Organization of Animal Health, 2020).

According to the ASEAN Good Animal Husbandry Practice Guidelines (ASEAN GAHP), pig farms should be located in areas with an appropriate clean water source, with physical barriers to reduce the risk of contamination arising from visitors and animal access. Quarantine pens should be located in a separate area and must be prepared for sick pigs and pig carcass to limit pathogen spread. Housing and equipment providing good hygiene and ventilation with easy-to-clean pig manure systems are recommended, along with good personal hygiene management for workers, including a pre-entry farm protocol which includes facilities for showering and hair washing, and changing into protective farm clothes and footwear before accessing the animal housing. Wastewater and manure should be kept in a closed area well away from the animal house, and the farm should possess a treatment system that limits odor and hazards such as biogas. Moreover, a veterinarian must authorize a program of vaccination and antimicrobial and chemical use for disease prevention and treatment. Vaccination is a potential method to prevent certain infectious diseases and is established as an important alternative to antibiotics (Founou et al., 2016). In many countries the governments produce and promote vaccines against the Foot and Mouth Diseases virus and Classical Swine Fever virus, as these are very important pathogens. While commercial and autogenous vaccines against pathogenic bacteria are alternatives to control infections, their efficacy varies considerably. To date, vaccines against bacteria such as *E. coli, Lawsonia intracellularis*, and *Actinobacillus pleuropneumonia* have been commercialized and used worldwide (FAO, 2014; Founou et al., 2016).

In 2013 the total amount of antimicrobials used for food-producing animal in SEA was 2,950 tons: antimicrobial classes included penicillin (666 tons), tetracyclines (484 tons), quinolones (321 tons), sulfonamides (317 tons) and macrolides (281 tons). Thailand consumed the largest amount of antimicrobials (531 tons in 2013), and the amount has subsequently increased (3,816.3 tons in 2018), probably due to the growing number of food-producing animals (Van Boeckel et al., 2017; Ministry of Public Health, 2020). In practice, antimicrobials are used for three indications: therapy, prevention, and growth promotion. Therapeutic use involves a short duration of treatment with a high dose of antimicrobial given only to sick pigs. Prophylactic administration is used to prevent disease occurring where it is routinely expected to occur (e.g., to control diarrhea after weaning), and the same amount and duration of administration is applied to all susceptible pigs during this period. Lastly, growth promotor use involves administering a lower than recommended therapeutic dose, long-term for all pigs on-farm (FAO, 2014). The intention is to improve growth rate by controlling subclinical infections and improving digestive efficacy. Antimicrobial growth promotion use has been banned in Thailand and Vietnam (Coyne et al., 2019), while Indonesia, Myanmar, and Timor-Leste policies promote a decrease in antimicrobial use in response to WHO announcements (Cardinal et al., 2020). Furthermore, the use of colistin as a feed additive has been restricted in Malaysia and Thailand (Olaitan et al., 2021), and nitrofuran has been banned in the Philippines and Thailand (Islam et al., 2014).

Implementation of standard biosecurity and management systems in pig farms is an essential part of veterinary preventive medicine, and promotes improved pig production, and reduced antimicrobial consumption in pigs, leading to fewer AMR bacteria on pig farms which otherwise might enter the food chain (Founou et al., 2016).

## Antimicrobial Usage and Relationship to Antimicrobial Resistance

The key driver of resistance in bacterial population is considered to be the extensive use of antimicrobial agents which created a selection pressure on susceptible bacteria (Aarestrup et al., 2008; Koningstein et al., 2010; Lunha et al., 2020). Frequency, amount, duration, and combined use of antimicrobial agents are important factors related to the emergence of both ARB and MDR bacteria (McEwen, 2006; Martinez and Baquero, 2009). Additionally, there have been many reports on a positive association between the amount of antimicrobial use and the prevalence of ARB in both animals and human (Lazarus et al., 2015). A recent study from Thailand reported the detection of high frequencies of ARB isolates from pigs, particularly resistance against tetracycline, which is also reported as an antimicrobial agent that has had long term and extensive use in livestock both in Thailand and throughout SEA (Van Boeckel et al., 2017; Lunha et al., 2020). Nevertheless, it is very important that monitoring of AMU using standardized systems for monitoring programs for AMR data is continued, and that the quantification and temporal trends in AMU are continued to be recorded in order to obtain evidence between the linkage of AMU and AMR so as to further support policy and decision makers to understand and fight against AMR (Aarestrup, 2005; Magouras et al., 2017).

## Detection of AMR and Clonal Analysis

At present, the main approaches to detect antibiotic resistance bacteria (ARB) and antibiotic resistance genes (ARG) from bacteria in hosts and environments are primarily categorized into the conventional culture-based methods, and molecular biology-based methods. Clone typing by core-gene multilocus sequence typing (MLST) is used to determine the molecular epidemiology of bacteria in the family *Enterobacteriaceae*.

### Conventional Culture-Based Methods

This approach requires isolating and growing the microorganisms of interest on a nutrient medium. For samples from wastewaters and associated environments the membrane filtration method is frequently applied along with selective media supplemented with the antibiotics of interest for isolation, and enumeration of individual ARB (Rizzo et al., 2013). The antibiotic resistance pattern of individual pure isolates can be further investigated by performing antibiotic susceptibility testing, which depends on the phenotypic traits of individual isolates. The frequently applied resistance susceptibility testing methods are of four types, including diffusion methods (Stokes method and Kirby-Bauer method), dilution methods (Minimum inhibitory concentration such as broth and agar dilution), diffusion and dilution methods (*E*-test method and gradient diffusion method), and automated instrument methods (such as Vitek 2 system by bioMérieux and Sensititre ARIS 2X by Trek Diagnostic Systems) (Jorgensen and Ferraro, 2009; McLain et al., 2016; Benkova et al., 2020). The results from antibiotic susceptibility testing should be interpreted following standardized values, which allow qualitative assessment distinction between susceptible, intermediate, and resistant isolates (Clinical & Laboratory Standards Institute: CLSI Guidelines; EUCAST: AST of bacteria; Jorgensen and Ferraro, 2009).

Nevertheless, the conventional culture-based methods are time-consuming and somewhat labor-intensive. Investigators are required to constantly attain a reliable and up-to-date interpretation of the resistance susceptibility testing. Besides, only a minority of bacteria are culturable and suitable for the conventional culture-based methods, which are presumed to be only a tiny fraction of the total bacteria present (Kummerer, 2004; Bouki et al., 2013; Rizzo et al., 2013).

### Molecular Biology-Based Methods

This approach allows the identification of deoxyribonucleic acid (DNA) targets with and without the requirement to culture individual isolates. Methods such as Polymerase Chain Reaction (PCR) amplification and use of DNA probes are well known for detecting resistance genes and genetic elements. With the application of multiplex PCR, various ARGs can be screened and detected simultaneously (Luby et al., 2016; Anjum et al., 2017; Preena et al., 2020). In comparison, quantitative PCR (qPCR) has been applied as a quantifying method for tracking and tracing ARGs from various pathogenic bacteria in different environments from municipal wastewater to surface water. However, the nucleic acid extraction method and its product quality are critical for PCR gene detection, since wastewater and sludge samples contain much indeterminate substance (Obst et al., 2006; Lupo et al., 2012; Rizzo et al., 2013). Additionally, several typing methods are used for determining and characterizing the diversity of resistant strains and clones of individual isolates, such as pulsed-field gel electrophoresis (PFGE), restriction fragment length polymorphism (RFLP), random amplified polymorphic DNA (RAPD), and amplified ribosomal DNA restriction analysis (ARDRA) (Hamelin et al., 2006; Faria et al., 2009; Li et al., 2009; Brooks et al., 2014; Papadopoulos et al., 2016). Unfortunately, there is limited availability of specific primer sets, especially for PCR and qPCR methods. These molecular techniques often encounter an absence of standardization and validation of interlaboratory tests (Rizzo et al., 2013).

Most recently, molecular high throughput (HPT) and next-generation sequencing (metagenomic analysis, transcriptome, and resistome) are among the techniques that facilitate studies of the microbial community and their diverse genomes and gene expression without a requirement for culture. Moreover, these techniques are very valuable since they can compute numerous target genes without knowledge of the bacteria or genes in the samples, are not limited by specific primer sets, and are even faster to perform than the conventional molecular methods such as PCR and qPCR (Rizzo et al., 2013; Limayem et al., 2019; Lira et al., 2020). They also provide a deeper insight into antibiotic resistance conditions related to both vertical and horizontal gene transfer occurrences (Von Wintersdorff et al., 2016).

Therefore, molecular biology-based methods make possible the detection of ARB and ARG that cannot be cultured and express in individual isolates and provide faster detection of ARB and ARG with high specificity and sensitivity compared with conventional culture-based methods. Nevertheless, these methods cannot separate between living and non-living microorganisms. They are also less useful for identifying ARB and resistance patterns compared to the conventional culture-based methods.

### Clonal Analysis of AMR Strains

Bacterial strains identified using DNA fingerprinting techniques can be assigned as clones representing the ancestor’s phylogenetic root tree. In general, bacteria are highly diverse genetically, even at the species-level, because of inter-and intraspecies gene interchange leading to diversification of genotype in a particular niche of a bacterial clone (a clonal complex). For AMR, clonal relations are used as a tool to indicate the phenomenon of bacterial transmission or “spillover” between sources: humans to animals, animals to the environment, or environment to humans or vice versa (Spratt, 2004). The diversification of bacterial clones depends on the extent of gene recombination and varies by bacterial type. Some species, e.g., *Salmonella enterica* members, are stable clones, whereas in some species, e.g., *Helicobacter pylori*, these may be transient or genetical diverse without phenotypical change (Selander et al., 1990; Kennemann et al., 2011). Index genetic parameters are used to assign the clones and clonal complexes. In the 1980s to 2000s, the best results were achieved using pulsed-field gel electrophoresis and multilocus sequence typing (Schwartz and Cantor, 1984; Maiden et al., 1998). Since the Whole Genome Sequencing (WGS) era, WGS-based strain typing has been increasingly used to analyze bacterial pathogens and AMR bacteria in the public health field and for implementing disease control strategies (Snitkin et al., 2012; Harris et al., 2013; Walker et al., 2013). Core genome MLST (cgMLST) has become a standard tool for WGS-based strain typing as it has high accuracy and epidemiological concordance. Outcomes from cgMLST are analyzed by comparison of shared common genes sets from genomes within the same species, which can be used to determine the source and routes of transmission, trace cross-contamination of healthcare-associated pathogens, and identify antibiotic-resistant lineages or subpopulations (Dekker and Frank, 2016; Mellmann et al., 2016; Nadon et al., 2017). From each species-specific cgMLST scheme, there is the joint-calculated parameter called the “relatedness threshold.” If cgMLST allelic mismatches between two bacterial stains are less than (or equal to) the relatedness threshold, there are clonal relations between them (Miro et al., 2020). The relatedness threshold is calculated using an algorithm in the Ridom SeqSphere + online package (Calibrating the cgMLST Complex Type Threshold^1^). The relatedness threshold of clonality from cgMLST diversity depends on insight of species-specific population genetics (i.e., the relative impact of mutation and recombination on genetic variation) (Schurch et al., 2018), as shown in Table 1.

**TABLE 1:** Relatedness criteria used for the cgMLST schemes for clinically relevant bacteria.

Species	Relatedness threshold cgMLST alleles	References
*Acinetobacter baumannii*	≤8	Higgins et al., 2017
*Campylobacter coli, C. jejuni*	≤14	Cody et al., 2013; Llarena et al., 2017
*Enterococcus faecium*	≤20	de Been et al., 2015
*Escherichia coli*	≤10	Dekker and Frank, 2016
*Klebsiella pneumoniae*	≤10	Snitkin et al., 2012
*Salmonella enterica*	≤4	Vincent et al., 2018
*Staphylococcus aureus*	≤24	Bartels et al., 2015

For clonal relationships, the horizontal genetic transmission of mobile genetic elements (plasmids, integrative conjugative elements, transposons) and their relatedness are important parameters indicating the transmission of AMR characteristics (Conlan et al., 2014, 2016; Sheppard et al., 2016). There are not many reports of clonal typing during AMR surveillance in a One Health approach. Epidemiological data and sources of bacterial strains defining clonal relationships in Thailand and Vietnam are shown in Table 2; however, insights into bacterial clonal transmission from the production process to consumers and patients remain incomplete.

**TABLE 2:** Lists of clonal relationships of AMR strains from South East Asia Countries in the One Health paradigm.

Year	Countries	Bacteria	Source and epidemiological data	Molecular typing	Conclusion of genetic relativeness	References
2018	Thailand	*E. coli*	Isolates from pig in the finishing period and pork from the same pigs	PFGE, MLST, plasmid replicon typing	Similar pulsotypes and sequence type (ST) from 2 pairs of strains from pig and pork.	Lugsomya et al., 2018b
					Similar plasmid Inc group (Frep-HI2 and FIB-FIC-Frep) from 2 pairs of strains from pig and pork.	
2019	Thailand	*E. coli*	Isolates from pigs and healthy humans from the same province	MLST	4 STs of ESBL producing *E. coli* shared between humans and pigs (ST10, ST70, ST117, ST685).	Seenama et al., 2019
2019	Thailand	*Salmonella* Rissen	Isolates from pigs, pork from slaughterhouses and markets from the same region	cgMLST,	A close similarity (percentage was not defined) from cgMLST between pig and pork sources from slaughterhouses and markets.	Prasertsee et al., 2019
2020	Thailand	*Salmonella* Weltevreden	Isolates from beef from a fresh market and isolates from patients in the same province	cgMLST, *in silico* plasmid typing	A high similarity from cgMLST between patient and beef sources.	Patchanee et al., 2020
					Same plasmid oriT type (MOB F) and plasmid incompatibility group (IncFII) of *mcr-1* plasmids from patient and beef strains.	
2021	Vietnam	*E. coli*	Isolates from pigs, chickens and patients	MLST, *in silico* plasmid typing	No evidence of clonal transmission between strains from a different host.	Nguyen et al., 2021
					The IS6 elements flanking ISEcp1–*bla*CTX-M–orf477/IS903B structures shared between strains from different hosts.	

## Possibility of AMR Transfer From Pigs to Humans

Humans can be exposed to antibiotic-resistant bacteria, both pathogenic and commensal bacteria, by direct transmission or through food or the environment. AMR problems are global issues since resistant bacteria can occur from any sector and spread intra-species, inter-species, and across borders (Kempf et al., 2013; Daniel et al., 2015). Certain antibiotics, particularly the Critically Important Antimicrobials to human medicine are applied in livestock farming. Inappropriate use of antibiotics in animals accelerates selective pressure and is a cause of antimicrobial cross-resistance in human medicine (Marshall and Levy, 2011). Moreover, the genes encoding antibiotic resistance in bacteria from animals can be transferred to bacteria that are pathogenic to humans (Archawakulathep et al., 2014). Many studies have reported the emergence of ARB from pigs and spread to humans; for example, quinolone resistant Salmonella spreading from pigs to human in Taiwan, and nourseothricin resistant *E. coli* from pigs to pig farmers, families, municipal communities, and patients with urinary tract infections (Witte, 1998; Aarestrup, 2005). Since livestock farms are a hotspot for resistant bacteria, workers on farms and in the food chain are potentially exposed to AMR bacteria from animals.

## AMR in Livestock

The AMR situation is critical in LMICs, including in the SEA. Although there is social and economic progress in those regions, the average population is still in poverty (Singh, 2017). Moreover, inadequate sanitation, poor quality control in slaughterhouses, and conditions where people live adjacent to animals (backyard farming) accelerate the spreading of resistant pathogens and ARG (Cook et al., 2017). Self-medication is cheaper in those regions, and antibiotics are easily accessible over the counter, leading to an increase in antimicrobial use not only by farmers but also by people without proper diagnosis (Nguyen et al., 2013). The major potential pathways for spreading of resistant bacteria and/or genes between food animals and humans could be horizontal transmission or clonal transfer of resistant bacteria (Lipsitch et al., 2002; Chang et al., 2015). An association between clonal types of human cephalosporin-resistant *E. coli* with those from food animals linked through food products has been reported (Lazarus et al., 2015).

Additionally, a study in Denmark found the possible transmission of sulfonamide resistant *E. coli* via the food chain to a healthy human who did not receive antibiotic therapy (Hammerum et al., 2006; Wu et al., 2010). The occurrence of resistant bacteria carrying mobile genetic elements in livestock or food of animal origins is concerning. The spread of antibiotic-resistance genes via horizontal gene transmission could lead to acquisition of multidrug-resistant (MDR) bacteria. MDR infections in humans can cause treatment failure and high mortality, especially if resistance is to a drug of last resort. Worrisomely, there are growing cases of colistin-resistant *Enterobacteriaceae* and sporadic cases of carbapenem-resistant cases in pigs from some Asian countries.

A study in Vietnam found that almost half of the pig farms in Bac Ninh province had colistin-resistant *E. coli*, and farmworkers also harbored such bacteria (Dang et al., 2020). Moreover, colistin-resistant *E. coli* have been detected in humans from rural areas of Vietnam (5–71.4%) (Trung et al., 2017; Kawahara et al., 2019). The *mcr* genes are currently globally distributed and are mostly detected from livestock origins followed by from humans and meat products. In SEA, *mcr*-1 positive *E. coli* has been detected in Cambodia, Vietnam, Malaysia, Singapore, and Thailand (Olaitan et al., 2016; Paveenkittiporn et al., 2017; Runcharoen et al., 2017; Srijan et al., 2018). On the other hand, *K. pneumoniae* isolates harboring *mcr-*1 genes have been reported in Laos and Singapore (Rolain et al., 2016; Teo et al., 2016). Moreover, ESBL-producing *K. pneumoniae* clones ST307, ST2958 and ST2959 that were resistant to β-lactams, aminoglycosides, fluoroquinolones, macrolide, lincosamide and streptogramins, rifampicin, sulfonamides, trimethoprim, phenicols and tetracycline, have been observed to be shared between both pig and human-sources within and across abattoirs. *K. pneumoniae* has been suggested to be a potential unheeded reservoir of resistance in the food chain that impacts on public health (Founou et al., 2018).

In addition to enteric bacteria, livestock-associated methicillin resistant *Staphylococcus aureus* (LA-MRSA) is another resistant bacterium of substantial health concern. LA-MRSA has been found worldwide, predominantly among people involved in livestock farming (Smith et al., 2009; Frana et al., 2013). These bacteria can be transmitted to humans in proximity to MRSA-harboring animal skin, especially from pigs (Smith and Pearson, 2011). There are several reports regarding the occurrence of MRSA cases in Asian countries, including in Malaysia (5.5%), China (15%), and Northern Thailand (2.53%) (Patchanee et al., 2014). The spa type t4358-SCC*mec* V is the most abundant clone in Malaysia, whilst it is t337-SCCmec IX in Thailand. In northeastern Thailand patients were identified whose MRSA pulso type pattern (PFGE) was related to that of MRSA discovered in a diseased pig in the same area (Lulitanond et al., 2013). A similar case providing evidence for the potential for transmitting resistant clones from pigs to humans occurred where an isolate from a human and two isolates from pigs shared SCC*mec* IX-*S. haemolyticus* (Sinlapasorn et al., 2015). The occurrence of livestock associated ARB in SEA is shown in Table 3.

**TABLE 3:** Reported occurrence of livestock associated ARB in SEA.

Bacterial species	Antimicrobial resistance pattern	Livestock sources	Country	References
*Escherichia coli*	Tetracycline, trimethoprim/sulfamethoxazole, chloramphenicol, gentamicin, ampicillin, colistin	Swine	Thailand	Lugsomya et al., 2018b; Lunha et al., 2020
			Vietnam	Hounmanou et al., 2021; Nguyen et al., 2021
			Singapore	Guo et al., 2021
			China	Olaitan et al., 2016
*Escherichia coli*	Ampicillin, ciprofloxacin, gentamicin, colistin, ceftazidime	Poultry	Vietnam	Nguyen et al., 2021
*Escherichia coli*	Ceftazidime, cefotaxime, cefotaxime with clavulanic acid	Cattle	Malaysia	Kamaruzzaman et al., 2020
*Klebsiella pneumoniae*	Colistin, β-lactam, aminoglycosides, fluoroquinolones, phenicols, tetracycline, sulfonamides, trimethoprim, bleomycin	Swine	Laos	Rolain et al., 2016
			Singapore	Teo et al., 2016
			Malaysia	Mobasseri et al., 2021
Methicillin-resistant *Staphylococcus aureus*	Erythromycin, ceftriaxone, cefoxitin, ciprofloxacin, gentamicin, tetracycline, trimethoprim-sulfamethoxazole, clindamycin, quinupristin-dalfopristin, tigecycline, cephalexin, fusidic acid, oxytetracycline, penicillin	Swine	China	Cui et al., 2009
			Hong Kong	Guardabassi et al., 2009
			Malaysia	Neela et al., 2009; Khalid et al., 2009
			Thailand	Patchanee et al., 2014
*Salmonella* spp.	Ciprofloxacin, sulfisoxazole, phenicols, nalidixic acid, ampicillin, tetracycline, sulfamethoxazole trimethoprim, norfloxacin, ciprofloxacin, amoxicillin, chloramphenicol	Poultry	Malaysia	Goni et al., 2018
			Thailand	Prasertsee et al., 2019; Vidayanti et al., 2021
			Singapore	Zwe et al., 2018
			China	Xu et al., 2020
			Vietnam	Ha et al., 2012; Ta et al., 2014;
*Salmonella* spp.	Ampicillin, streptomycin, florfenicol, tetracycline, sulfamethoxazole/trimethoprim, gentamicin	Swine	China	Yi et al., 2017

Unfortunately, the role of farm animals in the emergence and dissemination of resistant bacteria or genes to humans is still controversial (Marshall and Levy, 2011). Further studies focusing on genomic data of resistant bacteria and nation-wide epidemiological approaches are required to explore the transmission of AMR from animals to humans.

## AMR in the Food Chain

As discussed, possible transmission routes of ARB and ARGs to humans may be from direct contact with colonized animals or from the consumption of foodstuffs that are contaminated with ARB/ARGs along the food chain (Founou et al., 2016; Thapa et al., 2020). There are different potential pathways for contamination of ARB and ARGs along the food chain. ARB may disseminate to crops and plants from the utilization of contaminated irrigation water, mostly from manure discharges of both human and animals and from irrigated soil (Gatica and Cytryn, 2013). Whilst livestock products such as pork, beef and poultry can be contaminated with ARB during the slaughtering processes, aquaculture products may become contaminate with ARB and ARGs that have already been circulating in aquatic environments (Bridier et al., 2019; Amarasiri et al., 2020). The phenotype of AMR bacteria may be influenced by food preservation stresses such as decreasing pH to under 5.0, or increasing salt to more than 4.5%, which has been shown to increase phenotypic antibiotic resistance in *E. coli, S. enterica* serovar Typhimurium, and *S. aureus* (McMahon et al., 2007). In addition, contamination by ARB and ARGs may occur during transportation and distribution of food products, or occur as a post-contamination event after food processing, or from a cross-contamination as a result of inappropriate food handlings which are among the most common contamination pathways along the food chain (Verraes et al., 2013; Holzel et al., 2018).

## AMR in Wastewater and Associated Environments

Alarmingly, there have been increasing reports about the existence of AMR in the environment, especially resulting from the activities of both humans and animal industries (Prestinaci et al., 2015). The concern focuses mainly on the increase in ARB and ARG resulting from the dissemination of antibiotics in the environment (Kraemer et al., 2019; Serweciñska, 2020). Wastewater has been suspected as being one of the hotspots for the spread and circulation of ARB and ARG (Rizzo et al., 2013; Pazda et al., 2019). Human and animal excreta have been demonstrated to contain large amount of antibiotics which are distributed to municipal wastewater where the antibiotics are absorbed into sewage sludge (Nagulapally et al., 2009; Singer et al., 2016). Sludge (biosolids) is produced from the wastewater treatment processes in the wastewater treatment plant, and contains a large amount of non-degraded fat, oil, and protein. This composition contributes to poor retrieval of water-soluble antimicrobial agents such as ciprofloxacin from sludge (Gerba and Pepper, 2009; Singer et al., 2016; Serweciñska, 2020). Therefore, it is no surprise that ARB and ARG have been detected mainly in samples from wastewater and associated environments such as groundwater and surface water (Barancheshme and Munir, 2018; Amarasiri et al., 2020; Sabri et al., 2020).

## AMR in the Slaughtering Process

The risk of AMR bacterial contamination in the food chain is associated with AMR commensal bacteria circulating on carcasses at slaughterhouses (EFSA, 2016). In addition, the direct exposure of workers to resistant bacteria can happen throughout the production cycle to the slaughtering process and during food preparation (You et al., 2016). The exposure of slaughterhouse workers to extended spectrum beta lactamase (ESBL)-producing *Enterobacteriaceae* has received global attention (Wadepohl et al., 2020). Substantial data relating to the association between ESBL producing *Enterobacteriaceae* in pigs, farmworkers and slaughterhouse workers has been reviewed (Alali et al., 2008; Hammerum et al., 2014; Huijbers et al., 2014; Dohmen et al., 2015). In the Netherlands, workers were most likely to be exposed to ESBL producing *E. coli* with *bla*_CTX–M–1_ in the early slaughtering process (before chilling of carcasses), at a prevalence of 4.8% (Dohmen et al., 2017). In comparison, a 4.5% prevalence of ESBL/AmpC producers was found in residents living in the vicinity of livestock farms in the Netherlands (Wielders et al., 2017). Exposure to ESBL carriage is especially high in the slaughtering process, particularly during removal of visceral organs and handling of the throat area where there is massive colonization with enteric bacteria (Lowe et al., 2011; Lăpuşan et al., 2012). Moreover, a high percentage (50%, *n* = 59) of MDR *E. coli* have been recovered from slaughterhouse workers, where the rates in Nigeria were more elevated in butchers than in cleaners (Aworh et al., 2021). In slaughterhouses in Thailand, ESBL-producing *E. coli* were detected in 76.7% of pig feces, 33.3% of fresh pork samples, and 75% of workers (Boonyasiri et al., 2014). In a comparative study between Thailand and Cambodia, the prevalence of ESBL-producing *E. coli* in pig rectal and carcass swabs were similar, at only 5.3–5.6%. Among these, ESBL-producing *E. coli* containing *bla*_TEM–1_ and *bla*_CMY–2_ were found in both countries, but the *bla*_CTX–M–15_ gene was detected only in the Thai isolates (Trongjit et al., 2016).

The occurrence of multidrug-resistance bacteria circulating in slaughterhouses also has been reporting in many studies in SEA. *Salmonella* spp. are frequently used as an indicator of pathogen and AMR carriage, reflecting management standards. In Thailand, salmonella isolates with high levels of resistance to tetracycline, ampicillin, sulfonamide-trimethoprim, and streptomycin, at approximately 63–82%, can be found in pigs, workers, and the environment from farms to slaughterhouses (Tadee et al., 2015; Phongaran et al., 2019). *Salmonella* Rissen was found to be a common serovar in a comprehensive survey of pig farms, slaughterhouses, and retail outlets in Northern Thailand (Phongaran et al., 2019). Moreover, Wu et al. (2019) compared AMR salmonella contamination in pig rectal swabs and carcasses between slaughterhouses with and without a hazard analysis critical control point (HACCP) system. Both systems had a high level of indirect contamination, but the HACCP system decreased salmonella contamination in carcasses and reduced the number of AMR patterns (Wu et al., 2019). In a comparison between pig slaughterhouses in Thailand and Laos, the prevalence of salmonella contamination of pig carcasses in the two countries were 30.9 and 53.3%, respectively. *Salmonella* spp. from both countries were highly resistant to sulfonamide (98.3%), ampicillin (91%), and tetracycline (92.5%). These isolates contained class1 integrons carrying resistance cassette *dfrA12-aadA2*. Interestingly, five *Salmonella* isolates from Thai pig carcasses were producers of extended-spectrum □-lactamase (ESBL) and harbored *bla*_CTX–M–14_, whereas isolations from Laos were negative. The study reflected differences in the hygienic procedures of slaughterhouses and antibiotics uses in the two countries (Sinwat et al., 2016). In Vietnam, salmonella isolated from pork slaughterhouses and retail shops were highly resistant to ampicillin, tetracycline, and chloramphenicol (Nguyen D. T. et al., 2016), and *Salmonella* Rissen isolated from a pig carcass in 2013 showed phenotypical colistin resistance, with a plasmid-mediated *mcr-1* gene (Gonzalez-Santamarina et al., 2020). In the Philippines, 46.7% of pig tonsil and jejunum samples from three below-standard slaughterhouses were positive for *Salmonella enterica*, rates higher than those from five national accredited slaughterhouses, although there was no significant difference in prevalence. Most isolates (*n* = 183) were resistant to nitrofurantoin (93.4%), ampicillin (67.8%), and sulfa-trimethoprim (80.3%) (Calayag et al., 2017), even though nitrofurantoin use in livestock had been banned for a decade. However, it needs to be determined whether the source of contamination was from farming practices or occurred in the slaughtering process. The occurrence of ARB in the slaughtering process is summarized in Table 4.

**TABLE 4:** Phenotypic AMR in organisms isolated from difference sources.

Reference	Country	Organism	Host	Source	No. sample	% positive of sample	Prevalence of AMR
Boonyasiri et al., 2014	Thailand	*ESBL producing E. coli*	Pig rectal swab	Farm	400	76.70%	
			Carcass swab	Slaughterhouse	18	33.30%	
			Pork	Market	15	61.50%	
			Stool of workers	Farm	30	77.30%	
			Stool of workers	Food-factory	544	75.50%	
Trongjit et al., 2016	Thailand	*Escherichia coli*	Carcass swab	Slaughterhouse	88	85.20%	AMP (87%), TRI (82%), SUL (67%), and 5.7% (10/175) was ESBL-producing *E. coli* that contained bla_CTX–M15,_ bla_TEM–1_, bla_CMY–2_
			Carcass swab/Pork	Fresh market	87	35.60%	
			Pig rectal swabs	Slaughterhouse	85	ND	
	Cambodia	*Escherichia coli*	Carcass swab	Slaughterhouse	20	55%	AMP (78%), TRI (64.3%), SUL (70.5%), and 4.5% (5/110) was ESBL-producing *E. coli* that some contained bla_TEM–1_, bla_CMY–2_
			Carcass swab/Pork	Fresh market	90	40%	
			Pig rectal swabs	Slaughterhouse	82	ND	
Tadee et al., 2015	Thailand	*Salmonella* spp.	Pigs	Farm	86	ND	TET (82.56%), AMP (81.40%), and STR (63.95%)
			Farm worker				
			Slaughterhouse worker	Slaughterhouse			
			Environment	Farm and slaughterhouse			
Phongaran et al., 2019	Thailand	*Salmonella* spp.	Pig feces	Slaughterhouse	562	37.54%	AMP (69.05%), TET(66.19%), and SXT (35.71%)
Wu et al., 2019	Thailand	*Salmonella* spp.	Pig rectal swab	Slaughterhouse	360	61.11% in HACCP, 17.22% in non-HACCP slaughterhouse	Rectal of HACCP; AMP (76.36%), CHL (11.82%), STR (60.00%), SXT (17.27%), TET (85.45%). Non-HACCP; AMP (61.29%), CHL (3.23%), STR (29.03%), SXT (38.71%), and TET (64.52%)
			Carcass swab		360	12.78% in HACCP, 36.67% in non-HACCP slaughterhouse	Carcass swab of HACCP;AMP (95.65%), STR (95.65%), TET(95.65%).Non-HACCP;AMP (69.70%), CHL (1.52%), STR (45.45%), SXT (15.15%), and TET (69.70%)
Sinwat et al., 2016	Thailand	*Salmonella* spp.	Pig rectal swab	Slaughterhouse	185	34%	AMP (100%), TET (91.4%), STR (74.3%), SPE (87.1%), SUL (100%), TRI (55.7%)
			Carcass swab	Slaughterhouse	184	30.90%	AMP (96.7%), TER (91.8%), STR (75.4%), SPE (86.9%), SUL (100%), TRI (52.5%) and 5 isolates was ESBL-producing strains with bla_CTX–M–14_
			Carcass swab	Fresh market	180	60.50%	AMP (96.4%), TET (91.4%), STR (81.3%), SPE(80.6%), SUL(100%) and 8 isolates was ESBL-producing strains with blaCTX-M-14
			Worker	Slaughterhouse	52	13.40%	AMP (68%), TET (84%), STR (68%), SPE (88%), and SUL (100%)
			Butchers	Fresh market	50	4%	
			Patients	Hospital	78	17.90%	
	Laos	*Salmonella* spp.	Pig rectal swab	Slaughterhouse	129	38.70%	AMP (95%), TET (89.8%), STR (84.7%), SPE (57.6%), and SUL (91.5%)
			Carcass swab	Slaughterhouse	137	53.30%	AMP (91.5%), TET (97.6%), STR (96.3%), SPE (67%), and SUL (100%)
			Carcass swab	Fresh market	112	72.30%	AMP (91.3%), TET (93.8%), STR (96.9%), SPE (69.8%), SUL (95.8%), and TRI (56.6%)
			Worker	Slaughterhouse	36	25%	AMP (62.5%), TET (100%), STR (87.5%), SPE (81.3%), SUL (100%), and TRI (62.5%)
			Butchers	Fresh market	8	0.00%	
			Patients	Hospital	36	11.10%	
Nguyen N. T. et al., 2016	Vietnam	*Salmonella* spp.	Pork	Slaughterhouse	30	69.70%	AMP (54.2%), TET(627%), and CHL (508%)
				Retail stores	69		
Gonzalez-Santamarina et al., 2020	Vietnam	*Salmonella* spp.	Pig carcass	Slaughterhouse	1	ND	AMP (blaTEM-1b), GEN and TOB [aac(6”)-Iaa, aac(3)-IId, ant(3”)-Ia], and COL (mcr-1)
Calayag et al., 2017	Philippines	*Salmonella* spp.	Pig tonsil	Slaughterhouse	240	46.7% of total, 44.0% of accredited slaughterhouse, 46.7% of locally registered slaughterhouse	NI (93.4%), AMP(67.8%), and SXT(80.3%)
			Jejunum		240		

*ND, no data; AMP, ampicillin; AUG, amoxicillin-clavulanic acid; CHL, chloramphenicol; CIP, ciprofloxacin; CTX, cefotaxime; NA, nalidixic acid; NOR, norfloxacin; STR, streptomycin; SXT, sulfamethoxazole-trimethoprim; TE, tetracycline; SPE, spectinomycin; SUL, Sulfamethoxazole; TRI, Trimethoprim; GEN, gentamicin; TOB, tobramycin; COL, colistin; NI, Nitrofurantoin.*

Overall, the risk of bacterial contamination in the slaughtering process and of pig products is critical, and there is still a lack of comprehensive monitoring throughout the system. Operation under HACCP programs and good manufacturing and sanitation practice (GMP) are intended to control meat safety (Samelis, 2006; Wu et al., 2019), but a reduction in salmonella and AMR bacteria prevalence is not relevant to its implementation in SEA. Decontamination applications post-slaughter aim to reduce bacterial contamination in pork, and include physical applications such as hot water washing, chilling, freezing, and high-pressure processing. Secondly, chemical applications such as treatment with organic acid solutions, or electrolyzed or ozonated water are recommended. Lastly, biological applications include bacteriophages and wrapping film with postbiotics containing lactic acid bacteria. However, all applications must take into account meat quality and the physiology of the end product (Bolumar et al., 2020; Li et al., 2020; Shafipour Yordshahi et al., 2020; Aydin Demirarslan et al., 2021).

## Alternatives to Antibiotics

The use of antibiotics on farms increase the possibility of the AMR transmission from livestock production systems to consumers. There have been many attempts to replace antibiotic use on farms. This review outlines two applications for reduction of antibiotic use, including for growth promotion and disease prevention. An overview of possible antibiotic alternatives for growth promotion derived from previous studies is presented in Supplementary Table 1 (Cha et al., 2012; Kim et al., 2014; Yoon et al., 2014; Zeng et al., 2014; Devi et al., 2015; Gois et al., 2016; Hossain et al., 2016; Lee et al., 2016; Peng et al., 2016; Sbardella et al., 2016; Tang et al., 2016; Upadhaya et al., 2016; Wan et al., 2016; Lan et al., 2017; Lei et al., 2017; Ma et al., 2017; Pan et al., 2017; Wu et al., 2017; Huang et al., 2018; Lei et al., 2018; Samolińska et al., 2018; Sayan et al., 2018; Seo et al., 2018; Xu et al., 2018; Pei et al., 2019; San Andres et al., 2019; Wang et al., 2019; Duarte et al., 2020; Petry et al., 2020; Ren et al., 2020; Satessa et al., 2020; van der Peet-Schwering et al., 2020; Wei et al., 2020; Zhang et al., 2020; Sun et al., 2021; Wang et al., 2021). These vary considerably from chemical compounds, biologically active substances, and microbially derived products. These could perform several functions, including eliminating pathogenic microbes, modulating gut microbial communities, strengthening intestinal integrity, enhancing growth performances, or diminishing morbidity from other causes (Cheng et al., 2014; Liao and Nyachoti, 2017; Zeineldin et al., 2019).

Although these alternatives could be used as antibiotic replacements in terms of in-feed growth promotion, they are not suitable for therapy. In addition, proper disease prevention measures, including vaccination and enhanced biosecurity, are needed to help reduce antibiotic usage. Vaccines trigger a protective immunity that simulates the effects of a natural infection without the negative consequences. Vaccination has been successfully used in animals (Rose and Andraud, 2017). In livestock production, vaccination has been extensively used for disease prevention caused by pathogenic bacteria or viruses, and as such it is an important substitute for antibiotic usage (Meeusen et al., 2007). Remarkably, even protection against viral infections may reduce antibiotic use by decreasing the risk of misdiagnosis and treatment of secondary bacterial infections (Potter et al., 2008). For example, vaccination against Porcine Circovirus Type 2 (PCV-2), which is an immune-suppressive viral infection leading to secondary bacterial infections, resulted in a significant diminution of antibiotic use in swine farms (Raith et al., 2016). Similarly, vaccination against Porcine Reproductive and Respiratory Syndrome (PRRS) virus on pig farms reduced antibiotic consumption (Van Looveren et al., 2015). Amongst important bacterial pathogens of swine, vaccination against *Lawsonia intracellularis*, which causes severe ileitis, has been reported to reduce oxytetracycline medication in pigs (Bak and Rathkjen, 2009), whilst immunization against the respiratory pathogen *Actinobacillus pleuropneumoniae* also reduced administration of antibiotics (Kruse et al., 2015).

Biosecurity and farm management are essential parts of disease prevention that can enhance health status and significantly diminish the risk of exposure of pigs to pathogens (Postma et al., 2016b). These practices have been applied in varied species, production systems, and for different pathogens (Dahiya et al., 2006). Practices such as all-in all-out housing systems, reduced stocking rates, increased ventilation and improved waste management all help to improve the health status of farms. Improvements in biosecurity have been widely accepted as an effective tool for protecting from introduction of diseases into farms (Lewerin et al., 2015). Postma et al. (2016a) found that better biosecurity resulted in a lower antibiotic usage from birth to slaughter in pig herds. Similarly, Raasch et al. (2018) and Caekebeke et al. (2020) reported the same relationship between increased biosecurity in pig farms and reduced antibiotic use.

In summary, various means are available to reduce antibiotic use on farms. The addition of some alternatives and improved disease prevention in practice are important tools to reduce antimicrobial usage in swine farm and support global policy. Their increased use in the SEA region is to be encouraged.

## Policy and Strategies on AMR in Food Animals in the Association of South East Asian Nation (Asean)^2^

The importance of AMR as a human security threat has been recognized worldwide since the 1990s. In response the World Health Organization arranged a series of consultative meetings and developed recommendations for action. The culmination of this work was the 2001 WHO global strategy for containment of AMR (Sack et al., 2001). Later, in 2015, the Global Action Plan on AMR (GAP-AMR) was adopted by WHO member states at the World Health Assembly, the Food and Agricultural Organization Governing Conference and the World Assembly of World Organisations for Animal Health (World Health Organization [WHO], 2015). Member States committed to develop multi-sectoral national action plans on AMR and endorsed a ‘One Health’ approach to facilitate collaboration among various sectors and actors in the defense of human, animal, and environmental health. Through global political declarations, a common direction to tackle AMR was seen across countries and sectors (United Nations [UN], 2016).

Most countries in the ASEAN region have taken action to tackle AMR through the development of their National AMR action plan. According to the Global Database for the 2019, Tripartite AMR Country Self-assessment Survey (TrACSS), all eleven countries in the ASEAN region have developed a National action plan (NAP) which the government has approved (World Health Organization [WHO], 2018). The One Health approach has been adopted as a key principle for multi-sectoral coordination, and it was enshrined in all NAPs (Chua et al., 2021). Other policies for controlling and optimizing the use of antimicrobials have been developed through legal provisions and program implementation, including surveillance of AMR and antimicrobial consumption in both humans and the animal sectors.

Reviews of policy interventions that address AMR in food animal production indicate that there are many existing regulations for controlling veterinary medicines, including antimicrobials, in ASEAN countries. However, they are relatively varied across countries (Goutard et al., 2017). Policies of banning antibiotics for growth promotion in food animals have been enforced in some countries, such as Singapore and Thailand (Chua et al., 2021) while others have yet to enforce the ban. Thailand has restricted the use of antimicrobials for growth promotion since 2015 (Sommanustweechai et al., 2018). In 2019, some antimicrobial classes were assigned the highest priority, with critically important antimicrobials for human medicine only being available for food animals through veterinary prescription (notably polymyxins, third and fourth generation cephalosporins, macrolides, and fluoroquinolones) (Scott et al., 2019). However, a few SEA countries, including Vietnam and Myanmar, have neither laws nor regulations on the prescription and sale of antimicrobials for animal use (World Health Organization [WHO], 2018). In terms of antimicrobial stewardship programs, NAPs in most countries in the Region cover both human and animal health sectors, except for Cambodia and Malaysia. Indonesia and Thailand set codes of practices for control of veterinary drug use. Likewise, Brunei has set up national guidelines for prudent use of antimicrobial in livestock (Goutard et al., 2017).

Surveillance on AMR and antimicrobial consumption is a vital component of GAP-AMR. The WHO Global Antimicrobial Resistance and Use Surveillance System (GLASS) and Organisation for Animal Health global database on antimicrobials in animals were established to help provide evidence on the emergence of resistance and trends in antimicrobial use globally (World Health Organization [WHO], 2017). The 2019 TrACSS reported that most countries in the ASEAN region have a national surveillance system for AMR in food animals, with only a few countries such as Laos and Myanmar having inadequate laboratory capacity to perform AMR surveillance. Only Thailand and Malaysia have established systems for monitoring antimicrobial consumption in animals, in which are recorded data on the total quantity of antimicrobials sold for/used in animals nationally, by antimicrobial class, by species, method of administration, and by type of use. Malaysia and Thailand share their reports with the OIE annually (Chua et al., 2021). Thailand has successfully published One Health Reports on AMR, antimicrobial consumption, and public knowledge and awareness on AMR in 2017 and 2018, contributing to an evidence-based policy decision (NSC, 2020a, b). The 2019 version will be launched in April 2021.

Despite existing national commitments, implementation of AMR policy remains a significant challenge, particularly with the monitoring of AMR and AMU in the human and animal sectors. The monitoring systems are hampered by lack of institutional capacity, inadequate investment in human resources, and the need to strength data platforms for routine monitoring. Significant financial boosts are required to support these areas.

## Conclusion

This paper addressed the cross-species transmission of AMR and ARGs in the SEA. The importance of pig production in SEA has encouraged particular attention to this industry, but similar problems occur with other animal industries (chicken, fish etc.). Due to the limited number AMR studies, it is difficult to quantify the extent of the problem in the region, although some countries have better data available than others. Certainly, there is reason to believe that transmission of resistant bacteria and ARGs from pigs at the farm and through the slaughtering process provide risks to the human population, either through direct contact or via contaminated environments or pork products. Efforts should be made to reduce antimicrobial use at the farm level by improving farm hygiene and the use of alternatives to antibiotics. The AMR policy in the region has been implemented, although progress in this area has varied considerably between different countries due to different implementation’s capacities. Additional funding to support AMR surveillance and improved control in SEA is required.

## Author Contributions

NP: original idea, study design, and supervision of writing. WS: AMR distribution in food chain, wastewater, and detection. PA and PP: alternatives to antibiotics and data analysis. AL: policy and strategy controlling AMR in SEA. IS and NK: risk of AMR in pig production cycle and slaughtering. KL: molecular epidemiology analysis. DH: study design, supervision, and proofreading of the writing. All authors contributed to the article and approved the submitted version.

## Conflict of Interest

The authors declare that the research was conducted in the absence of any commercial or financial relationships that could be construed as a potential conflict of interest.

## Publisher’s Note

All claims expressed in this article are solely those of the authors and do not necessarily represent those of their affiliated organizations, or those of the publisher, the editors and the reviewers. Any product that may be evaluated in this article, or claim that may be made by its manufacturer, is not guaranteed or endorsed by the publisher.

## Footnotes

1
https://www.ridom.de/u/Server_Database_Initialization.html

2 The scope of this article covers 11 member countries of the Association of Southeast Asian Nations (ASEAN). They are: Brunei Darussalam, Cambodia, Indonesia, Lao PDR, Malaysia, Myanmar, the Philippines, Singapore, Thailand, and Vietnam.

## Supplementary Material

Supplementary Table 1 Summary of available alternatives to antibiotics in swine production.
