# Supplementary material for: Reducing the Risk of Transmission of Critical Antimicrobial Resistance Determinants From Contaminated Pork Products to Humans in South-East Asia
Source: Front Microbiol. 2021 Jul 27;12:689015. doi: 10.3389/fmicb.2021.689015 (PMC8353453; doi:10.3389/fmicb.2021.689015)
Supplement: Supplementary Table 1 — Summary of available alternatives to antibiotics in swine production. [file Table_1.docx]

SUPPLEMENTARY MATERIAL

**Reducing the risk of transmission of critical antimicrobial resistance determinants from contaminated pork products to humans in South-East Asia**

**Wandee Sirichokchatchawan^1^, Prasert Apiwatsiri^2^, Pawiya Pupa^2^, Imporn Saenkankam^2^, Nwai Oo Khine^2^, Angkana Lekagul^3^, Kittitat Lugsomya^4^, David J. Hampson^5^ and Nuvee Prapasarakul^2,6*,^**

^1^ College of Public Health Sciences, Chulalongkorn University, Bangkok, Thailand.

^2^ Department of Veterinary Microbiology, Faculty of Veterinary Science, Chulalongkorn University, Bangkok, Thailand

^3^ International Health Policy Program, Ministry of Public Health, Nonthaburi, Thailand

^4^ Jockey Club College of Veterinary Medicine and Life Sciences, City University of Hong Kong, Kowloon Tong, Hong Kong Special Administrative Region.

^5^ School of Veterinary Medicine, Murdoch University, Perth, Western Australia

^6^ Detection and Monitoring of Animal Pathogens Research Unit, Chulalongkorn University, Bangkok, Thailand.

* Correspondence:

Nuvee Prapasarakul

Nuvee.p@chula.ac.th

**Supplementary Table 1** Summary of available alternatives to antibiotics in swine production

| Active ingredient | Conc. | Period | Pathogen challenge | Growth promotion | | Gut microbial composition | | Gut integrity | | Clinical signs reduction | References |
| --- | --- | --- | --- | --- | --- | --- | --- | --- | --- | --- | --- |
|  |  |  |  | Weight gain | Feed efficiency | Lactobacilli | *E. coli* and Coliforms | Morphology | Barrier |  |  |
| In-feed enzymes | | | | | | | | | | | |
| Phytase | 20,000 FTU/kg diet | Weaned | - |  |  | - | - | - | - | - | (Zeng et al., 2014) |
|  | 500 FTU/kg diet | Nursery | - |  |  | - | - |  | - | - | (Ren et al., 2020) |
| Lysozyme | 1 g/kg | Weaned | - |  |  | **↓** | **↓** | - |  | - | (Ma et al., 2017) |
|  | Lysozyme milk derived from transgenic sow | Neonate | ETEC O149:K88 (5×10^9^ CFU) | - | - | **↑** | **↓** |  |  |  | (Huang et al., 2018) |
| Xylanase | 0.005-0.01% | Weaned | - |  |  | **↑** |  | - | - |  | (Lan et al., 2017) |
|  | 100 mg/kg | Grower | - |  |  | - | - |  |  | - | (Petry et al., 2020) |
| Zinc Oxide (ZnO) | | | | | | | | | | | |
| ZnO | 3000 ppm | Weaned | - |  |  |  | **↓** |  | - |  | (Pei et al., 2019) |
|  | 2400 ppm | Weaned | - |  |  |  | **↓** | - |  | - | (Wang et al., 2019) |
|  | 2500 ppm | Weaned | - |  |  | - | - |  | - |  | (Satessa et al., 2020) |
|  | 2500 ppm | Nursery | - |  |  | **↓** | - | - | - | - | (Wei et al., 2020) |

Significant effect No effect **↑** Significant increase **↓** Significant decrease - Not available

**Supplementary Table 1** Continued

| Active ingredient | Conc. | Period | Pathogen challenge | Growth promotion | | Gut microbial | | Gut integrity | | Clinical signs reduction | References |
| --- | --- | --- | --- | --- | --- | --- | --- | --- | --- | --- | --- |
|  |  |  |  | Weight gain | Feed efficiency | Lactobacilli | *E. coli* and Coliforms | Morphology | Barrier |  |  |
| Organic acids | | | | | | | | | | | |
| 0.2% Blended organic acids | 17% fumaric acid, 13% citric acid, 10% malic acid, and 1.2% capric acid and caprylic acid | Grower | - |  |  | **↑** |  | - | - | - | (Upadhaya et al., 2016) |
| 0.2-0.4% Blended organic acids | 17% fumaric acid, 13% citric acid, 10% malic acid, and 1.2% capric acid and caprylic acid | Weaned | *E. coli* K88  (5×10^9^ CFU) |  |  | - | - | - | - |  | (Lei et al., 2017) |
| 1.5 g/kg Blended organic acids | 50% benzoic acid, 3% calcium formate, 1% fumaric acid | Weaned | - |  |  | **↑** |  |  | - |  | (Xu et al., 2018) |

**Supplementary Table 1** Continued

| Active ingredient | Conc. | Period | Pathogen challenge | Growth promotion | | Gut microbial | | Gut integrity | | Clinical signs reduction | References |
| --- | --- | --- | --- | --- | --- | --- | --- | --- | --- | --- | --- |
|  |  |  |  | Weight gain | Feed efficiency | Lactobacilli | *E. coli* and Coliforms | Morphology | Barrier |  |  |
| Phytochemicals (Essential oil) | | | | | | | | | | | |
| *Phlomis umbrosa Turcz*, *Cynanchum wilfordii Hemsley*, *Zingiber officinale Rosc*, and *Platycodi Radix* | 0.05- 0.10% | Grower | - |  |  | - | - | - | - | - | (Devi et al., 2015) |
| Brazilian red pepper essential oil | 500 mg/kg | Weaned | - |  |  |  |  |  | - | - | (Gois et al., 2016) |
| Hop (*Humulus lupulus*) β-acids | 360 mg/kg | Weaned | - |  |  |  |  |  | - |  | (Sbardella et al., 2016) |
| 30 mg/kg Blended essential oil | thymol 25% and carvacrol 25% | Weaned | - |  |  | **↑** |  |  | - |  | (Xu et al., 2018) |
| Antimicrobial peptide (AMP | | | | | | | | | | | |
| AMP-A3 and AMP-P5 | 60 mg/kg | Weaned | - |  |  | - | **↓** |  | - | - | (Yoon et al., 2014) |
| Porcine β-defensin 2 (rpBD2) | 5 g/kg | Weaned | - |  |  | - | - |  |  | - | (Peng et al., 2016) |
|  | 0.1 mg/mL | Weaned | ETEC O149:K88 (10^9^ CFU) |  |  | **↑** | **↓** |  | - | - | (Tang et al., 2016) |
| Recombinant plectasin | 60 mg/kg | Weaned | - |  |  | **↑** |  |  |  |  | (Wan et al., 2016) |

**Supplementary Table 1** Continued

| Active ingredient | Conc. | Period | Pathogen challenge | Growth promotion | | Gut microbial | | Gut integrity | | Clinical signs reduction | References |
| --- | --- | --- | --- | --- | --- | --- | --- | --- | --- | --- | --- |
|  |  |  |  | Weight gain | Feed efficiency | Lactobacilli | *E. coli* and Coliforms | Morphology | Barrier |  |  |
| Bacteriophages | | | | | | | | | | | |
| ETEC specific lytic phage CJ12 (10^6^-10^8^ PFU/g) | 0.1% | Weaned | ETEC JG280 (10^10^ CFU/ml) |  |  | - | **↓** | - | - |  | (Cha et al., 2012) |
| Bacteriophage cocktails (10^9^ PFU/g) | 1 g/kg | Grower | - |  |  | **↑** | **↓** | - | - | - | (Kim et al., 2014) |
|  | 0.1% | Weaned | - |  |  | **↑** | **↓** |  | - |  | (Lee et al., 2016) |
| Lytic bacteriophage cocktail (10^9^ PFU/g) | 5 ml | Weaned | *Salmonella Typhimurium* ATCC 14028 (10^8^ CFU/ml) |  |  | - | - | - | - |  | (Seo et al., 2018) |
| Probiotics | | | | | | | | | | | |
| Bacillus *licheniformis* (1.5×10^10^ CFU/g) and *Saccharomyces cerevisiae* (0.3×10^10^ CFU/g) | 500 mg/kg | Weaned | ETEC K88  (10^9^ CFU/ml) |  |  | **↑** | **↓** |  | - |  | (Pan et al., 2017) |
| *Lactobacillus salivarius* (10^9^ CFU/ml) | 2 ml for 3 days and 5 ml for 7 day | Suckling | F4^+^ ETEC  (10^8^ CFU/ml) |  | - | **↑** |  |  | - |  | (Sayan et al., 2018) |
| *Bacillus amyloliquefaciens* DMS 25840 and *Bacillus subtilis* DSM 32324 | 6×10^8^ CFU/kg | Grower-finisher | - |  |  | - | - | - | - |  | (van der Peet-Schwering et al., 2020) |
| *Bacillus subtilis* and *Enterococcus faecium* | 2×10^8^ CFU/g | Weaned | - |  |  | - | - | - | - |  | (Zhang et al., 2020) |

**Supplementary Table 1** Continued

| Active ingredient | Conc. | Period | Pathogen challenge | Growth promotion | | Gut microbial | | Gut integrity | | Clinical signs reduction | References |
| --- | --- | --- | --- | --- | --- | --- | --- | --- | --- | --- | --- |
|  |  |  |  | Weight gain | Feed efficiency | Lactobacilli | *E. coli* and Coliforms | Morphology | Barrier |  |  |
| Probiotics | | | | | | | | | | | |
| *Bacillus subtilis* ms1, *Bacillus licheniformis* SF5-1, and *Saccharomyces cerevisiae* | 1.5×10^9^ CFU/g per each | Grower | - |  |  |  |  | - | - | - | (Wang et al., 2021) |
| *Lactobacillus acidophilus, Lactobacillus casei,*  *Bifidobacterium thermophilum* and *Enterococcus faecium* | 0.25×10^8^ CFU/g per each | Weaned | F18^+^ ETEC  (2×10^9^ CFU) |  |  | - | - |  | - |  | (Sun et al., 2021) |
| Prebiotics | | | | | | | | | | | |
| Lactulose | 0.5-1 g/kg | Weaned | - |  |  | **↑** | **↓** | - | - |  | (Hossain et al., 2016) |
| Isomalto-oligosaccharides | 6 g/kg | Weaned | - |  |  | - | - |  | - |  | (Wu et al., 2017) |
| Chicory (a prebiotic compounds such as insulin and oligofructose) and GroBiotic-S (prebiotic mixture of brewer’s yeast, dairy ingredient components, and dried fermentation products) | 0.05-0.5%  and  2.5% | Weaned | - |  |  | - | - | - | - | - | (San Andres et al., 2019) |

**Supplementary Table 1** Continued

| Active ingredient | Conc. | Period | Pathogen challenge | Growth promotion | | Gut microbial | | Gut integrity | | Clinical signs reduction | References |
| --- | --- | --- | --- | --- | --- | --- | --- | --- | --- | --- | --- |
|  |  |  |  | Weight gain | Feed efficiency | Lactobacilli | *E. coli* and Coliforms | Morphology | Barrier |  |  |
| Synbiotics (probiotic + prebiotic) | | | | | | | | | | | |
| [probiotic]  *Lactococcus lactis* IBB500, *Carnobacterium divergens* S1, *Lactobacillus casei* LOCK 0915, *Lactobacillus plantarum* LOCK 0862 (10^9^ CFU/g per each), and *Saccharomyces cerevisiae* LOCK 0141 (10^7^ CFU/g)  [prebiotic]  Inulin | 0.5 g/kg  (probiotic)  20 g/kg  (prebiotic) | Grower | - |  |  | - | - | - | - | - | (Samolińska et al., 2018) |
| [probiotic]  *Bacillus subtillus*, *Rhodopseudomonas capsulata* (10^10^ CFU/kg per each), and *Clostridium butyricum* (10^9^ CFU/kg)  [prebiotic]  Fructooligosaccharide | 1 g/kg  (probiotic)  1 g/kg  (prebiotic) | Grower | - |  |  | **↓** |  | - | - | - | (Lei et al., 2018) |
| [probiotic]  *Bacillus* sp.  [prebiotic]  Xylanase | 10^8^ CFU/kg  (probiotic)  10,000 XU/kg  (prebiotic) | Weaned | *E. coli* F18^+^  (6×10^9^ CFU) |  |  | - | - |  | - |  | (Duarte et al., 2020) |
